# The interplay of self-acceptance, social comparison and attributional style in adolescent mental health: cross-sectional study

**DOI:** 10.1192/bjo.2023.594

**Published:** 2023-10-27

**Authors:** Qian-Nan Ruan, Guang-Hui Shen, Jiang-Shun Yang, Wen-Jing Yan

**Affiliations:** Psychological Counselling and Treatment Centre, Seventh People's Hospital, Wenzhou, China; School of Mental Health, Wenzhou Medical University, Wenzhou, China; and Zhejiang Provincial Clinical Research Centre for Mental Illness, Affiliated Kangning Hospital, Wenzhou Medical University, Wenzhou, China

**Keywords:** Adolescents, self-acceptance, social comparison, attributional style, mental health

## Abstract

**Background:**

Adolescence is a pivotal stage vulnerable to mental health problems such as anxiety and depression. Although self-acceptance and social comparison are known to affect adolescent mental health, their interactive and moderating roles are not fully understood.

**Aims:**

To explore the role of self-acceptance, social comparison and attributional style in predicting these mental health outcomes among adolescents in clinical settings.

**Method:**

A cross-sectional study was conducted on a sample of 242 adolescents. Participants completed measures assessing self-acceptance, social comparison, attributional style and mental health outcomes (depression and anxiety). Mediation models and multi-group analysis were used to examine the relationships among these variables.

**Results:**

Our findings demonstrated a significant relationship between self-acceptance, social comparison, depression and anxiety (*r*s = 0.32–0.88). Specifically, lower self-acceptance and higher social comparison were associated with higher levels of depression and anxiety. Additionally, individuals with external attributional tendencies reported higher depression (Cohen's *d* = 0.61) and anxiety (*d* = 0.58) compared with those with internal tendencies. Mediation modelling showed that social comparison is a mediator between self-acceptance and depression (effect size −0.04, 95% CI −0.08 to −0.01) and anxiety (effect size −0.06, 95% CI −0.10 to −0.02). Crucially, multi-group analysis showed that the impact of social comparison on mental health outcomes varied significantly based on attributional style.

**Conclusions:**

These findings underscore the importance of considering self-acceptance, social comparison and attributional style in understanding and addressing mental health challenges during adolescence. This could inform the development of targeted interventions to promote mental health and well-being among adolescents. However, further research is needed to confirm these findings in diverse populations and to explore the underlying mechanisms in greater detail.

## Adolescence: a vulnerable developmental phase

Adolescence is a crucial developmental phase characterised by notable physical, emotional and cognitive transformations, which makes young people particularly susceptible to mental health problems, namely anxiety and depression.^1^ Recent estimates indicate that each year about 20% of adolescents worldwide experience mental health problems, mostly depression and anxiety, with these conditions sometimes persisting into adulthood.^2^ Given the significant impact of these disorders on adolescents’ quality of life, academic performance and future psychosocial adjustment,^3^ there is a critical need for a deeper understanding of their aetiology and contributing factors.

During adolescence, individuals often experience an intensified focus on self-identity, self-concept and self-acceptance.^4^ Furthermore, adolescence marks a time when social comparison becomes particularly salient, partly owing to increased significance of peer relationships and a heightened awareness of social hierarchies.^5^ These evolving dynamics around self-perception and social evaluation intersect with broader psychosocial elements, such as attributional styles, that also undergo critical developmental shifts during adolescence. Therefore, understanding the complex relationships between self-acceptance, social comparison and mental health outcomes such as depression and anxiety becomes especially crucial at this stage.

## Self-acceptance, social comparison and attributional style

Self-acceptance and social comparison, which are both fundamental components of adolescents’ psychological functioning, have been identified as significant correlates of adolescent mental health.^6^ Self-acceptance, defined as an individual's acceptance of themselves, including their strengths and weaknesses, has been linked to better psychological well-being and lower levels of anxiety and depression.^7^ Self-acceptance is understood as a fundamental component of psychological health, as it relates to an individual's ability to embrace their unique qualities, including strengths and weaknesses.^8^ One study^9^ suggested that self-acceptance is integral to mental health, as individuals with higher levels of self-acceptance are typically more resilient in the face of stress and adversity. Moreover, research has consistently demonstrated a negative correlation between self-acceptance and mental health problems such as depression and anxiety.^6^ For instance, one study^10^ revealed that individuals with low self-acceptance levels tend to experience more negative emotions, including depressive and anxious symptoms, signifying the protective role of self-acceptance against mental health problems.

On the other hand, social comparison, defined as the process of comparing oneself with others, has been linked to psychological distress. Festinger^11^ proposed the social comparison theory, suggesting that individuals inherently have a drive to assess their abilities and opinions, which is often done by comparing themselves with others. The negative psychological effects of social comparison can be especially potent during adolescence, a developmental period characterised by heightened peer comparison and approval-seeking behaviours.^12^ Research suggests that unfavourable social comparison can lead to feelings of inferiority, envy and dissatisfaction, which may increase the risk of developing symptoms of depression and anxiety.^13,14^

These relationships could be complicated by other individual differences, such as attributional style. Attributional style theory, initially proposed by Weiner,^15^ suggests that the way people explain the causes of events, as being internal or external, has a significant impact on their emotional well-being. This theory has been supported by several studies. For instance, Peterson et al^16^ found that children and adolescents who made more internal attributions (attributing events to factors within themselves) for negative events were more likely to exhibit symptoms of depression. Further, research conducted by Sweeney et al^17^ suggested that individuals who attribute negative events to external factors – outside of their control – tend to report higher levels of stress and anxiety. This is consistent with the learned helplessness theory, which suggests that people who consistently attribute negative events to uncontrollable factors may begin to feel helpless, leading to depression and anxiety.^18^ A study by Kleim et al^19^ showed that trauma survivors with higher levels of external attributions for their trauma were more susceptible to developing post-traumatic stress disorder, a type of anxiety disorder. Similarly, a meta-analysis conducted by Joiner & Wagner^20^ found a significant association between external attributional style and depressive symptoms across numerous studies.

## Bridging the research gap: an integrated perspective

Despite the substantial body of research showing the influence of self-acceptance and social comparison on mental health, notably depression and anxiety, a conspicuous gap exists in our understanding of the complex interplay among these variables, particularly during the crucial period of adolescence. This gap is twofold.

First, most studies to date have treated self-acceptance and social comparison as separate influences on mental health, with limited research investigating the potential mediating role of social comparison in the relationship between self-acceptance and mental health outcomes. Studies have established that higher self-acceptance is associated with better mental health^21,22^ and that negative social comparison can lead to feelings of inferiority and subsequent mental health problems.^11,13^ However, the relationship between these variables remains unclear. For instance, does low self-acceptance lead to negative social comparison, which in turn contributes to depression and anxiety? Or do low self-acceptance and negative social comparison independently contribute to these mental health problems? A deeper understanding of these relationships could lead to more effective interventions, by identifying key targets (e.g. promotion of self-acceptance, teaching healthier social comparison strategies) that could alleviate adolescent depression and anxiety.

Second, there is a dearth of research investigating how these relationships may be moderated by individuals’ attributional tendencies. Attributional style, or how individuals interpret the causes of events, has been linked with various mental health outcomes, including depression and anxiety.^18,23^ Specifically, an external attributional style – attributing outcomes to factors outside oneself – has been associated with feelings of helplessness and increased mental health problems.^18^ It is plausible that adolescents with an external attributional style may be particularly prone to the negative effects of low self-acceptance and negative social comparison. For instance, individuals with an external attributional style may be more likely to engage in detrimental social comparisons and have lower self-acceptance, thereby increasing their risk of mental health consequences.^13^ Conversely, those with an internal attributional style, who attribute events to their own actions, may engage in more adaptive social comparison strategies and demonstrate higher levels of self-acceptance, thus potentially protecting against depression and anxiety.^24^ However, to the best of our knowledge, no study has yet examined the potential moderating function of attributional style in these associations.

Given the developmental stage under investigation, we contend that self-acceptance serves as the primary mover. As adolescents negotiate their evolving identities, self-acceptance becomes the cornerstone of their intrapsychic world, providing the lens through which they interpret their self-worth. Identity formation is at the forefront during this life stage.^25^ It is this internal state of self-acceptance that then influences the manner and extent to which adolescents engage in social comparison. Hence, we chose to model self-acceptance as the predictor and social comparison as the mediator, grounded in developmental psychology theory. To address these gaps, it is critical to conduct studies that explore the mediating role of social comparison in the relationship between self-acceptance and mental health outcomes, while also considering the potential moderating effect of attributional style. Such research would not only contribute to the theoretical understanding of these constructs, but also potentially guide the development of targeted interventions to enhance mental health among adolescents.

## Method

### Participants

A total of 242 adolescent out-patients were selected by convenience to receive one-on-one mental health assessments. Prior to the mental health assessment, the patients and their parents/guardians were informed about the assessment procedure and scales to be used and both a parent/guardian and the patient provided written informed consent, as required by local hospital ethics regulations. The mean age of the participants was 14.96 years (s.d. = 1.54 years). The sample comprised 81 (33.47%) males and 161 (66.53%) females; 71 participants (29.34%) had a foster or ‘left-behind’ experience in their past; 57 participants (23.55%) were from incomplete families. In terms of attributional tendency, 147 participants (60.74%) had an internal attributional tendency and 95 (39.26%) had an external attributional tendency (Table 1).

**Table 1:** Sociodemographic information of the participants (*n* = 242)

	*n*	%	Mean	s.d.
Age, years			14.96	1.54
Gender				
Male	81	33.47		
Female	161	66.53		
Left-behind experience				
Left behind	71	29.34		
Not left behind	171	70.66		
Family structure				
Complete	185	76.45		
Incomplete	57	23.55		
Attributional tendency				
External attribution	95	39.26		
Internal attribution	147	60.74		
Self-acceptance			31.33	9.07
Social comparison			34.89	8.46
Depression			14.79	8.28
Anxiety			11.52	6.98

### Measures

#### Demographic information

Basic demographic information about each adolescent, such as gender, age, whether they had any experience of being left behind and family structure, were recorded. Gender (female versus male), left-behind experience (left-behind versus not left-behind), family structure (complete or incomplete) were coded as binary variables.

#### Attributional styles

The Multidimensional-Multiattributional Causality Scale (MMCS)^26^ was utilised to assess participants’ attributional styles related to achievement. The 24-item Achievement Locus of Control subscale was selected for this study. Each item is rated on a 5-point Likert scale from 0 (disagree) to 4 (agree), with higher scores indicating higher degrees of endorsement for a particular attributional tendency. Where an internal attribution score was greater than or equal to an external attribution score, this was coded as an internal attributional tendency, and vice versa. Previous research also found the MMCS to have good construct and convergent validity, with Cronbach's α = 0.83.^27^ In the present study, the Cronbach's α for the MMCS Achievement subscale was 0.84, indicating good reliability for use with this sample.

#### Self-acceptance

Self-acceptance was measured using the Self-Acceptance Questionnaire (SAQ), a 16-item measure comprising two subscales: Self-Acceptance and Self-Evaluation.^28^ The Self-Acceptance subscale includes eight reverse-scored items rated on a 4-point Likert scale from 1 (strongly disagree) to 4 (strongly agree). The Self-Evaluation subscale includes eight positively worded items, also rated on a 4-point Likert scale from 1 (strongly disagree) to 4 (strongly agree). Subscale scores were summed, with higher scores indicating greater self-acceptance. In the current study, the SAQ demonstrated good internal consistency, with Cronbach's α = 0.89.

### Social comparison

Social comparison was assessed using the 11-item Iowa–Netherlands Comparison Orientation Measure (INCOM),^14,29^ a widely used measure of individuals’ tendency to compare themselves with others. Each INCOM item is scored on a 5-point Likert scale from 1 (strongly disagree) to 5 (strongly agree). Higher total scores reflect a greater social comparison orientation. Previous research found the INCOM to be a reliable and valid measure of social comparison tendencies in diverse populations.^14^ In the present study, the INCOM demonstrated good internal consistency (Cronbach's α = 0.82).

#### Depression

Depressive symptoms were assessed using the Patient Health Questionnaire-9 (PHQ-9),^30^ a 9-item measure derived from the DSM-IV criteria for major depressive disorder. Each PHQ-9 item is rated on a 4-point Likert scale from 0 (not at all) to 3 (nearly every day), with total scores ranging from 0 to 27. Higher scores indicate greater severity of depressive symptoms. A total score ≥15 was used to identify cases of likely major depressive disorder, providing good specificity for diagnosis.^30^ Previous research shows the PHQ-9 to have good validity and reliability in both clinical and community populations. In the present study, the PHQ-9 demonstrated high internal consistency, with Cronbach's α = 0.92.

#### Anxiety

Generalised anxiety disorder symptoms were assessed using the 7-item Generalized Anxiety Disorder scale (GAD-7).^31^ The GAD-7 asks participants to rate how often they have been bothered by anxiety symptoms over the previous 2 weeks on a 4-point Likert scale from 0 (not at all) to 3 (nearly every day). Total scores range from 0 to 21, with higher scores indicating greater anxiety severity. A score ≥10 has been established as a cut-off for likely diagnosis of generalised anxiety disorder. Previous research shows the GAD-7 to have good reliability and validity for screening anxiety in clinical practice and research.^32^ In the present study, the GAD-7 showed excellent internal consistency, with Cronbach's α = 0.94.

### Statistical analysis

Statistical analyses were performed using SPSS Version 23.0 and R Version 4.2.1 for Windows. SPSS was used for initial data entry and exploratory analyses. R was used specifically for mediation modelling. First of all, descriptive statistics and reliability analyses were conducted. Second, independent samples *t*-tests compared scores on depression, anxiety, self-acceptance and social comparison measures across external and internal attribution subgroups. Separate *t*-tests were run for each variable, with α set at 0.05. Cohen's *d* was calculated as an estimate of effect size, with 0.20 constituting a small effect, 0.50 a medium effect and 0.80 a large effect.^33^ Third, mediation models were constructed to determine whether social comparison mediated the relationship between self-acceptance and depression/anxiety symptom severity. Direct and indirect effects were estimated using the product method, with 95% bias-corrected confidence intervals derived from 1000 bootstrap samples. Indirect effects were considered significant where the confidence interval did not cross zero. Finally, a multi-group structural equation model was constructed to test whether this mediation model differed significantly across external and internal attribution subgroups. Separate mediation models were specified for each subgroup, and model fit was compared to determine whether freeing or constraining certain pathways across groups resulted in significantly better fit using the χ^2^ difference test.^34^ A non-significant χ^2^ difference would indicate that the pathway could be constrained to be equal across groups; a significant χ^2^ difference would indicate that the pathway should be freely estimated, suggesting group differences. Significant differences in specific mediation pathways between external and internal attribution groups were then explored by calculating the critical ratio of difference (CR_d_). If the absolute value of the CR_d_ is higher than 1.965, then there is an intergroup difference at the level of *P* < 0.05.^34^

### Ethics approval and consent to participate

The authors assert that all procedures contributing to this work comply with the ethical standards of the relevant national and institutional committees on human experimentation and with the Helsinki Declaration of 1975, as revised in 2008. All procedures involving human subjects/patients were approved by the institutional review board of Wenzhou Seventh People's Hospital (EC-KY-2022048).

## Results

Descriptive statistics for the measurements of the 242 participants are shown in Table 2. Self-acceptance and social comparison were both significantly associated with anxiety and depression (*P* < 0.001), left-behind experience and family structure were significantly associated with depression (*P* < 0.05) and left-behind experience was significantly associated with anxiety (*P* < 0.05). Independent samples *t*-tests showed that individuals with external attributional tendencies reported higher levels of depression and anxiety than those with internal attributional tendencies. More specifically, it was found that depression scores were significantly higher in the external attribution group (mean 17.73, s.d. = 6.80) than in the internal attribution group (mean 12.90, s.d. = 8.61) (*t* = 4.61, *P* < 0.001, Cohen's *d* = 0.61). Anxiety scores were also significantly higher in the external attribution group (mean 13.91, s.d. = 5.88) than in the internal attribution group (mean 9.97, s.d. = 7.23) (*t* = 4.44, *P* < 0.001, Cohen's *d* = 0.58).

**Table 2:** Correlation analysis among study variables

	Age	Gender	Left-behind	Family structure	Self- acceptance	Social comparison	Depression	Anxiety
Age	1							
Gender	−0.08	1						
Left behind	−0.07	−0.11	1					
Family structure	−0.08	0.06	−0.07	1				
Self-acceptance	0.21**	−0.21**	0.14*	−0.05	1			
Social comparison	0.09	0.14*	−0.11	0.03	−0.32***	1		
Depression	−0.23***	0.23***	−0.18**	0.13*	−0.76***	0.33***	1	
Anxiety	−0.19**	0.23***	−0.16*	0.11	−0.73***	0.39***	0.88***	1

*P < 0.05, **P < 0.01, ***P < 0.001.

### Mediated model

Multiple regression analysis (Table 3) was conducted to examine the mediating role of social comparison in the relationships between self-acceptance and anxiety/depression. Age, gender, foster and left-behind experience, and family structure were included as covariates. The analysis revealed that social comparison was negatively predicted by self-acceptance (*β* = −0.33, *P* < 0.001), indicating that lower self-acceptance was associated with higher social comparison tendencies. Depression was negatively predicted by self-acceptance (*β* = −0.67, *P* < 0.001) and positively predicted by social comparison (*β* = 0.11, *P* < 0.01). Anxiety was negatively predicted by self-acceptance (*β* = −0.49, *P* < 0.001) and positively predicted by social comparison (*β* = 0.18, *P* < 0.001). A mediation analysis was conducted to assess whether social comparison mediated the relationships between self-acceptance and both depression and anxiety symptoms. The results supported that social comparison significantly mediated the links between self-acceptance and both depression and anxiety symptoms (Table 4). Self-acceptance had indirect effects on depression (effect size −0.04, 95% CI −0.06 to −0.02) and anxiety (effect size −0.06, 95% CI −0.10 to −0.02) through social comparison. Lower self-acceptance predicted higher social comparison, which in turn predicted higher levels of depression and anxiety symptoms.

**Table 3:** Regression analysis for mediated model

	Social comparison			Depression			Anxiety		
	*β*	s.e.	*P*	*β*	s.e.	*P*	*β*	s.e.	*P*
Age	0.10	0.04	0.01	−0.06	0.03	0.02	−0.04	0.03	0.17
Gender	0.15	0.13	0.24	0.11	0.10	0.25	0.11	0.10	0.27
Left behind	−0.09	0.13	0.49	−0.15	0.10	0.11	−0.10	0.10	0.32
Family structure	0.04	0.15	0.78	0.18	0.10	0.05	0.15	0.10	0.15
Self-acceptance	−0.33	0.07	<0.001	−0.67	0.04	<0.001	−0.49	0.03	<0.001
Social comparison	(–)	(–)	(–)	0.11	0.04	0.01	0.18	0.05	<0.001

*β*, standardised coefficient.

**Table 4:** Mediation model effect decomposition

Paths	Effect size	s.e.	*Z*	*P*	Bootstrapping 95% CI
Indirect effect					
Self-acceptance → Social comparison → Depression	−0.04	0.02	−2.15	0.03	−0.08 to −0.01
Self-acceptance → Social comparison → Anxiety	−0.06	0.02	−2.73	0.006	−0.10 to −0.02
Direct effect					
Self-acceptance → Depression	−0.68	0.04	16.44	<0.001	−0.76 to −0.60
Self-acceptance → Anxiety	−0.64	0.04	14.85	<0.001	−0.72 to −0.56
Total effect					
Depression	−0.71	0.04	18.94	<0.001	−0.79 to −0.63
Anxiety	−0.69	0.04	18.36	<0.001	−0.77 to −0.61

### Group difference test

To investigate whether the mediated relationships differed by attributional tendency, multi-group analyses were conducted (Table 5). The free model allowed all path coefficients to differ between external and internal attribution groups. The constrained model held path coefficients equal between groups. The result of chi-squared testing highlighted a significant difference between the free model and constrained model (depression: Δχ^2^_(3)_ = 8.40, *P* < 0.05; anxiety: Δχ^2^_(3)_ = 8.68, *P* < 0.05). This indicates that the path coefficients in the mediation model differed significantly between external and internal attribution groups. Path coefficients for the mediated model in each attribution group are presented in Table 5. CR_d_ were calculated to determine which paths differed significantly between groups. The paths from social comparison to depression (CR_d_ = 19.00, *P* < 0.05) and social comparison to anxiety (CR_d_ = 5.40, *P* < 0.05) had CR_d_ exceeding 1.96, indicating that these relationships were significantly stronger for adolescents with an internal attributional style than for those with an external style. For the internal attribution group, increased levels of social comparison were associated with greater symptoms of depression (*β* = 0.19, *P* < 0.001) and anxiety (*β* = 0.27, *P* < 0.001). In contrast, social comparison was not a significant predictor of depression (*β* = 0.01, *P* = 0.97) or anxiety (*β* = 0.05, *P* = 0.43) for the external attribution group.

**Table 5:** Paths and critical ratios for differences by attribution group

Model path	External attribution group			Internal attribution group			CR_d_
	*β*	s.e.	*P*	*β*	s.e.	*P*	
Self-acceptance → Social comparison	−0.27	0.12	0.02	−0.40	0.08	<0.001	1.48
Self-acceptance → Depression	−0.56	0.08	<0.001	−0.67	0.06	<0.001	1.20
Social comparison → Depression	0.01	0.06	0.97	0.19	0.06	0.001	19.00*
Self-acceptance → Anxiety	−0.54	0.08	<0.001	−0.62	0.06	<0.001	1.15
Social comparison → Anxiety	0.05	0.07	0.43	0.27	0.06	<0.001	5.40*

*β*, standardised coefficient; CR_d_, critical ratio of difference.

*P < 0.05.

### Mediation model effect decomposition

**Fig. 1:**
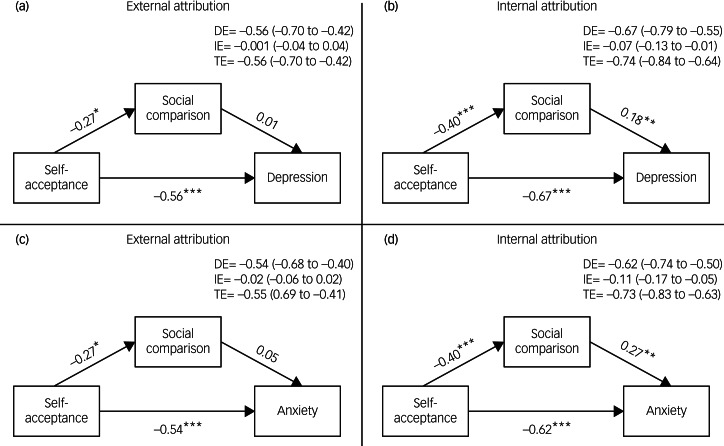
Mediation models examining the effects of self-acceptance on depression/anxiety through social comparison in the external attributional tendency group (a and c) and the internal attributional tendency group (b and d). DE, direct effect; IE, indirect effect; TE, total effect. Effects of each model are presented with 95% confidence intervals. *P < 0.05, **P < 0.01, ***P < 0.001.

To further explore the nature of relationships within the mediation model for each attribution group, effect decomposition was conducted (Fig. 1). Standardised direct, indirect and total effects of self-acceptance on depression and anxiety were estimated separately for external and internal attribution groups. In the external attribution group, for depression, the direct effect of self-acceptance was −0.56 (95% CI −0.70 to −0.42), indicating that lower self-acceptance significantly predicted higher depression. The indirect effect via social comparison was 0.001 (95% CI −0.04 to 0.04) and did not contribute significantly to the total effect. The total effect of self-acceptance on depression was −0.56 (95% CI −0.70 to −0.42). The same results held for anxiety, with a significant direct effect of self-acceptance on anxiety and a non-significant indirect effect via social comparison. For adolescents who have an external attributional style, the social comparison did not significantly influence the relationship between self-acceptance and symptoms of depression or anxiety. In the internal attribution group, both the direct effect of self-acceptance on anxiety and depression and the indirect effect through social comparison were significant.

## Discussion

The aim of our study was to investigate the connections among self-acceptance, social comparison and mental health outcomes (anxiety and depression) in adolescents. In addition, we aimed to assess the potential mediating role of social comparison and to understand how attributional styles may influence these relationships. Our results uncovered significant findings that have further enriched our understanding of these relationships. First, we found that lower self-acceptance was associated with a higher propensity for social comparison, which in turn was associated with more severe symptoms of anxiety and depression. Our mediation model analysis also suggests that social comparison is a mediating factor between self-acceptance and these poor mental health outcomes. Second, our findings revealed a key role of attributional styles in these associations. Specifically, individuals with external attributional tendencies reported significantly higher depression and anxiety than those with internal tendencies. This new insight regarding the influence of attributional styles on mental health outcomes and their interaction with self-acceptance and social comparison substantially expands on existing knowledge in the field.

### Interpretation of our findings and comparison with existing literature

Our initial crucial discovery indicated that adolescents exhibiting external attributional tendencies reported significantly higher levels of depression and anxiety compared with those with internal tendencies. This discovery resonates with the existing body of research in the field of attributional theory. For instance, Peterson & Seligman's^18^ groundbreaking work proposed that individuals with an external locus of control, often characterised by a belief that events in their lives are primarily influenced by external circumstances rather than their own actions, tend to exhibit higher rates of depression and anxiety. Our study provides further evidence supporting this theory, substantiating the concept that external attributional tendencies can be a significant predictor of mental health outcomes. Notably, the direct comparison between external and internal attributional styles in relation to anxiety and depression symptoms as demonstrated in our study adds a more nuanced understanding of this relationship and fills a gap in the literature.

The second central observation of our study was that lower levels of self-acceptance were associated with heightened tendencies for social comparison, which in turn were linked with increased symptoms of anxiety and depression. This finding broadens our understanding from the previous research focusing on the individual roles of self-acceptance and social comparison in mental health. A study conducted by Gilbert et al^35^ indicated that self-acceptance is inversely related to depressive symptoms. Meanwhile, separate research by Gibbons & Buunk^14^ confirmed that individuals with higher tendencies for social comparison often report elevated levels of anxiety and depression. By intertwining these two factors, our study contributes to the field by highlighting the combined influence of self-acceptance and social comparison on mental health outcomes. Such a significant relationship is of particular relevance during adolescence. This is a stage marked by an increased importance of peer relationships and social acceptance, and thus higher social comparison tendencies.^36^ Lower self-acceptance may therefore be particularly harmful in this context, exacerbating social comparison and leading to increased symptoms of anxiety and depression.

Perhaps one of the most remarkable discoveries of our research was that social comparison did not have a major mediating effect on the association between self-acceptance and depression or anxiety in individuals with an external attributional style. This observation diverges from conventional wisdom of social psychology that posits social comparison as a universal process affecting mental health, regardless of attributional style.^11,37^ A few theories offer potential insight. First, attributional styles could shape cognitive appraisals, thus influencing emotional and behavioural outcomes tied to self-acceptance and social comparison. For instance, adolescents with an external attributional style may perceive adverse social comparisons as outcomes beyond their control, thereby mitigating the impact of social comparison on their mental well-being.^18^ Second, learned helplessness theory^38^ posits that individuals with an external attributional style may develop a sense of helplessness, which could lessen the emotional toll of unfavourable social comparisons. Finally, self-determination theory^39^ proposes that the intrinsic versus extrinsic motivations governed by attributional styles might differently modulate how adolescents process self-acceptance and social comparison, thus leading to varied mental health outcomes.

### Implications and future research

Although our study has laid groundwork in illustrating the moderating role of attributional styles, further research is needed to empirically test these potential mechanisms. Our findings indicate that the connection between self-acceptance, social comparison and mental health outcomes may be more complex than previously thought, involving moderating factors such as attributional styles. This finding may have more implications for adolescents, because adolescence is a critical period for the development of stable attributional styles,^40^ suggesting that interventions aimed at promoting healthier attributional styles during this period may be particularly beneficial. Furthermore, the role of attributional styles in our study resonates with the research that adolescence is a formative period for the crystallisation of attributional tendencies. This developmentally relevant angle is underscored by the emergent importance of peer comparison during adolescence.^36^ Therefore, our study not only adds a nuanced layer to our understanding of self-acceptance and social comparison as independent variables but also enriches the broader discourse on psychosocial development during adolescence. This suggests that targeted interventions aimed at fostering healthier attributional styles and coping mechanisms related to self-acceptance and social comparison may be particularly effective during this developmental phase.

However, our study also had some findings that were not entirely conclusive, leaving room for further investigation. For instance, although our findings indicate that social comparison has a varying influence depending on attributional style, the specific mechanisms of this effect remain elusive. Therefore, future research could delve into this area, examining the potential underlying psychological or neurobiological factors that could explain this interaction. Another potential area for future research would be to explore the role of other individual differences, such as personality traits or resilience factors, in the relationships between self-acceptance, social comparison and mental health outcomes.

### Limitations

This study is subject to certain limitations. The cross-sectional design limits the ability to infer causality or directionality among the variables under study. A longitudinal approach would offer a more nuanced understanding of the temporal relationships between self-acceptance, social comparison, attributional style and mental health outcomes. Second, the data were collected using self-report measures, which are susceptible to biases, including social desirability. Future research could integrate alternative assessment methods, such as clinician evaluations or peer ratings, to triangulate the findings. Third, the sample is culturally specific, consisting solely of Chinese adolescents from clinical settings. This limits the generalisability of the findings to other cultural and demographic groups. Fourth, the use of convenience sampling from a clinical population narrows the scope of the study and may not fully represent the broader adolescent population. The application of these findings to community samples or other settings warrants caution. Consequently, future research should aim to replicate these findings in more diverse populations and settings to enhance external validity.

## Data Availability

The data-set analysed in the current study is available at https://1drv.ms/f/s!Ah0WQ_R767kBj1WjEICiSpYM5fzm?e=aJboEf.
